# Achieving the state of Georgia 25% HIV incidence reduction target among men who have sex with men in Atlanta through expanded use of multimodal pre-exposure prophylaxis: A mathematical model

**DOI:** 10.1371/journal.pone.0312369

**Published:** 2025-01-09

**Authors:** Jeremy Fraysse, Sarah-Jane Anderson, Justin C. Smith, Derrick D. Matthews, Supriya Sarkar, Filipa de Aragao, Rob Blissett

**Affiliations:** 1 ViiV Healthcare, Health Outcomes, The Netherlands; 2 GlaxoSmithKline, Value, Evidence & Outcomes, United Kingdom; 3 Campaign to End AIDS at Positive Impact Health Centers, Georgia, United States of America; 4 Harvard T.H. Chan School of Public Health, Boston, MA, United States of America; 5 Weitzman Institute, Moses/Weitzman Health System, United States of America; 6 ViiV Healthcare, Epidemiology & Real-World Evidence, Durham, NC, United States of America; 7 NOVA National School of Public Health, Public Health Research Centre, Universidade NOVA de Lisboa, Lisbon, Portugal; 8 Incremental Action Consulting, Lda, Lisbon, Portugal; 9 Maple Health Group, LLC, New York, United States of America; Centers for Disease Control and Prevention, UNITED STATES OF AMERICA

## Abstract

The US faces substantial demographic and geographic disparities in both HIV burden and access to pre-exposure prophylaxis (PrEP), an effective strategy to prevent HIV acquisition. Long-acting cabotegravir (CAB) is a novel, injectable PrEP option which demonstrated superior reduction in risk of HIV acquisition compared to daily-oral PrEP in the HPTN083 trial. We modelled the impact of increased PrEP initiations and the introduction of long-acting CAB on HIV incidence among men who have sex with men (MSM) in Atlanta, Georgia, a population with a high burden of HIV. The Georgia Department of Public Health has set an ambitious 25% HIV incidence reduction target, which could be reached with a daily-oral PrEP coverage of 42.2%. However, the target could be achieved at lower levels of PrEP coverage (34.6%) if a mix of PrEP modalities was used, such as an equal split of long-acting CAB PrEP and daily-oral PrEP. Our results clearly demonstrate that broadening access to new PrEP options has the potential to facilitate the achievement of public health HIV incidence reduction goals at plausible levels of PrEP coverage.

## Introduction

The number of new HIV diagnoses in the United States (US) remains high, with over 36,000 incident diagnoses in 2021, of which 66% were in men who have sex with men (MSM) [1]. Some communities are disproportionately impacted by the burden of new HIV diagnoses, with 40% of new diagnoses in Black/African Americans and 29% in Hispanic/Latinos, 56% of diagnoses in people aged 13 to 34, and 52% of diagnoses in the US South [1].

In 2019, the US Department of Health and Human Services proposed the ambitious Ending the HIV Epidemic (EHE) plan to reduce the number of new HIV infections nationally by 75% by 2025, and by at least 90% by 2030 [2]. A key strategy to achieve these targets is expanded use of biomedical HIV pre-exposure prophylaxis (PrEP), which enables individuals who are at elevated risk of HIV acquisition to lower that risk with the use of antiretroviral medications [2]. The EHE plan is supported by the National HIV/AIDS Strategy (2022–2025), which prioritizes expanded PrEP coverage in key populations with disproportionate burden of new HIV diagnoses [3].

Daily-oral PrEP options including tenofovir disoproxil fumarate with emtricitabine and tenofovir alafenamide with emtricitabine have been demonstrated to substantially reduce the risk of HIV acquisition when taken as prescribed [4]. While high adherence to daily-oral PrEP in MSM is required to achieve the highest levels of protection (4 or more pills taken per week) [4, 5], several attributes associated with daily-oral PrEP have been shown to limit initiations, adherence, and persistence. These include daily pill burden, lack of discretion leading to stigmatization, provider challenges in ensuring the continuity of prescriptions, and concerns regarding potential side effects and/or start-up symptoms [6–10].

Despite the availability of effective oral PrEP for over a decade, current coverage levels of daily-oral PrEP are not reaching national targets and, in 2021, only 30% of people who could benefit from PrEP received a prescription in the US [11]. Further, disparities in access to PrEP exist, with younger people and Blacks/African Americans experiencing the highest burden of new HIV diagnoses while being less likely to receive a PrEP prescription [12]. The Centers for Disease Control and Prevention (CDC) estimate that only 11% of Black/African American people who could benefit from PrEP were prescribed PrEP in 2021, compared with 78% of their White counterparts. This disparity in PrEP access has the potential to worsen current inequalities in the burden of new HIV diagnoses [11, 13].

Real-world studies of PrEP implementation programs focused on increasing daily-oral PrEP engagement in key MSM populations have been described in the literature [14–17]. These studies have focused on addressing challenges to PrEP engagement across different levels: individual, clinical encounters, and health system. A variety of approaches have been studied, for example behavioral interventions utilizing motivational interviewing [14, 15], culturally tailored counselling [16], and utilizing incentives and peer mentoring [17]. Encouraging findings indicate that with resources dedicated to PrEP implementation programs focused on addressing barriers to use, an approximate doubling of daily-oral PrEP initiations relative to standard of care is achievable [14–17].

Novel PrEP modalities have been sought to address the adherence-dependent efficacy of daily-oral PrEP. In 2021, a long-acting injectable option administered every 8 weeks, cabotegravir (CAB) PrEP, was approved by the Food and Drug Administration. Long-acting CAB PrEP demonstrated superior reduction in risk of HIV acquisition compared with daily-oral tenofovir disoproxil fumarate with emtricitabine in MSM and transgender women in the HPTN 083 trial [18]. Critically, long-acting CAB PrEP demonstrated consistently high adherence and efficacy across key populations that have reported low rates of adherence to daily-oral PrEP, including young adults and Black/African American MSM [18]. Furthermore, the availability of a long-acting PrEP modality may increase overall PrEP engagement. In a related area of sexual health, the introduction of long-acting modalities of contraception resulted in overall increases in engagement compared to the availability of daily-oral options only [19]. Studies of US MSM PrEP preferences have reported that 31–67% of MSM who currently or previously used daily-oral PrEP are interested in switching to long-acting PrEP and, importantly, that 25–47% of MSM not using PrEP would prefer a long-acting option over a daily-oral [20, 21].

Previous modelling studies have evaluated the impact of the introduction of long-acting PrEP on rates of HIV acquisition in priority regions such as the US South and cities such as Atlanta [22–24]. However, they did not evaluate PrEP expansion scenarios to estimate the increase in PrEP coverage that can be reasonably expected based on a single PrEP modality [24, 25], and certain studies have focused on scenarios involving only the use of daily-oral or long-acting PrEP without consideration of a multimodal PrEP mix [22, 26]. Furthermore, the modelled scenarios included high coverage of interventions (e.g. 100% antiretroviral therapy initiation for people living with HIV and 90% PrEP coverage in indicated individuals) that may not be achievable, and thus be of potentially limited value to public health decision makers [23, 27]. Finally, strategies to address barriers to PrEP use in key populations have not been included to compare with the introduction of new PrEP modalities.

In Georgia, the State Department of Public Health has set a target of a 25% HIV incidence reduction among all MSM, with a specific focus on expanding use of PrEP to achieve 50% PrEP coverage in indicated MSM [28]. The aim of the present study was to estimate, using a mathematical model, the impact of strategies to increase PrEP coverage and the resulting decreases in HIV incidence among MSM in Atlanta, GA, through 2030. Specifically, we assessed the potential to efficiently achieve local targets (25% reduction in HIV incidence) through expanding PrEP implementation programs that enhance uptake of daily oral PrEP and through the introduction of long-acting CAB PrEP to the PrEP modality mix in the Atlanta MSM community. The results from this analysis will help to inform public health decision making and effective allocation of scarce resources at the state and national level [29].

## Methods

This study expanded on a previously developed dynamic network model of HIV transmission (EpiModelHIV) calibrated to the demographics among MSM in Atlanta interacting within a sexual network “designed” by ARTnet [24].

### Model adaptation and validation

Prior work by Maloney et al. [24] was adapted to reflect updated demographic, programmatic, and clinical trial data. The adaptation process is described briefly below with further details available in S1 Appendix “Model updates” section, including Tables A and B in S1 Appendix.

The network model was re-estimated using data from the American Community Survey (ACS) five-year estimate (2018) [30] to describe the proportion of MSM by race (33.9% Black; 10.6% Hispanic; and 55.5% White/Other). The model’s HIV transmission factors for racial groups then required recalibration such that the steady state HIV prevalence by race matched that of published 2014 estimates [31]. Calibration was performed using an approximate Bayesian computation rejection scheme with manually tuned ad-hoc sequential steps. Calibration runs took place over 60 years. Once the transmission factors had been tuned, the model was run for 1,000 simulations of 10,000 agents (i.e., individuals) and the best fitting run was used to populate the model projection phase. Daily-oral PrEP was introduced in the model in 2016 with initiations of long-acting CAB PrEP starting in 2023. The demographics of the modelled cohort, as well as the burden of new HIV diagnoses and PrEP coverage were well aligned with real-world data against which the model was validated (Figs A-C in S1 Appendix).

PrEP utilization patterns, including uptake likelihood, adherence, and persistence, differ in the real-world setting based on individual demographic characteristics such as age, gender, and race [32]. However, in previous evaluations, modeled PrEP usage patterns were independent of demographic characteristics of MSM. Here, uptake, adherence, and persistence with daily-oral PrEP were specified for different demographic groups using data from large real-world studies of PrEP utilization patterns (including data provided by the authors of Tao, et al. 2020) [33, 34] and the CDC AtlasPlus database [35], which provides PrEP coverage rates by demographics and geography. The modelled long-acting CAB PrEP efficacy reflects data from the HPTN083 trial [18]. It was assumed that the efficacy is reflective of the compliance to dosing schedule observed in HPTN083 (91.5% coverage), which was found to be consistent across demographics in HPTN 083 subgroup analyses [18]. It was conservatively assumed that long-acting CAB PrEP persistence (i.e., period of continued use of the intervention) matched that of daily-oral PrEP, despite sub-optimal daily-oral PrEP persistence rates [2, 3], as real-world data on long-acting PrEP utilization patterns are not yet available. The updated model underwent validation against HIV incidence and prevalence estimates for Atlanta derived from CDC surveillance data, details of which are described in S1 Appendix “Model validation” section.

The resulting adapted model used in this study provides estimates of coverage (time on PrEP) overall and by demographic subgroups that is needed to achieve public health targets for HIV incidence reductions with an efficient allocation of limited healthcare resources. PrEP coverage levels (defined as person-years on PrEP divided by person-years with an indication for PrEP) were evaluated in the indicated MSM population and resulting HIV incidence outcomes in the full Atlanta MSM community.

### Scenarios analyzed

In this analysis, a series of scenarios was run, examining the impact of expanding the reach of PrEP implementation programs and the introduction of an additional long-acting PrEP option. The epidemiological impact of each scenario was measured starting in 2023 through 2030. These scenarios differed in probability of PrEP uptake and/or split of PrEP modality between daily-oral and long-acting PrEP. Results from the following scenarios are presented: 1) reference scenario (status quo): maintained 2021 levels of PrEP uptake with daily-oral PrEP only, 2) doubled probability of daily-oral PrEP initiations (proportional to current uptake patterns across demographic groups) to reflect expansion of PrEP implementation programs. In addition, a series of scenarios examining increased probability of PrEP uptake to achieve 25% HIV incidence reduction were evaluated: 3) with a 100:0 daily-oral : long-acting split (i.e., daily-oral PrEP only), 4) with a 75:25 daily-oral : long-acting split, 5) with a 50:50 daily-oral : long-acting split, 6) with a 25:75 daily-oral : long-acting split, and 7) with a 0:100 daily-oral : long-acting split (i.e., long-acting CAB PrEP only).

## Results

In the reference scenario based on current daily-oral PrEP coverage patterns, 44.3% of Black MSM, 48.2% of Hispanic MSM, and 54.0% of MSM aged ≤34 years had the highest levels of adherence to daily-oral PrEP, and therefore the highest levels of protection against HIV infection (Table 1).

**Table 1:** Age and race/ethnicity distribution across PrEP adherence levels in the model.

Age group	Black	Hispanic	White	Total
High adherence (≥4 daily oral PrEP pills per week)				
**Total**	44.3%	48.2%	54.0%	51.9%
**15 to 24**	30.9%	31.9%	38.8%	36.2%
**25 to 34**	41.0%	44.4%	48.2%	43.9%
**35 to 44**	46.5%	49.1%	54.6%	43.6%
**45 to 54**	49.1%	52.2%	57.8%	51.8%
**≥55**	49.4%	49.5%	57.8%	53.7%
Medium adherence (2–3 daily oral PrEP pills per week)				
**Total**	37.8%	36.6%	34.6%	35.3%
**15 to 24**	41.0%	41.0%	39.4%	39.9%
**25 to 34**	39.0%	38.1%	37.0%	38.2%
**35 to 44**	37.2%	36.4%	34.5%	38.0%
**45 to 54**	36.2%	35.3%	33.0%	35.5%
**≥55**	36.3%	36.1%	33.0%	34.8%
Low adherence (<2 daily oral PrEP pills per week)				
**Total**	18.0%	15.2%	11.4%	12.8%
**15 to 24**	28.1%	27.1%	21.8%	23.9%
**25 to 34**	19.9%	17.5%	14.9%	17.9%
**35 to 44**	16.4%	14.5%	10.9%	17.8%
**45 to 54**	14.7%	12.5%	9.1%	12.7%
**≥55**	14.3%	14.4%	9.1%	11.5%

PrEP, pre-exposure prophylaxis.

With current patterns of daily-oral PrEP use in Atlanta, PrEP coverage levels in indicated MSM were found to be 14.3% and HIV incidence reductions in the full Atlanta MSM community with current trajectory of PrEP use over the modelled period to be 3.1%.

**Fig 1:**
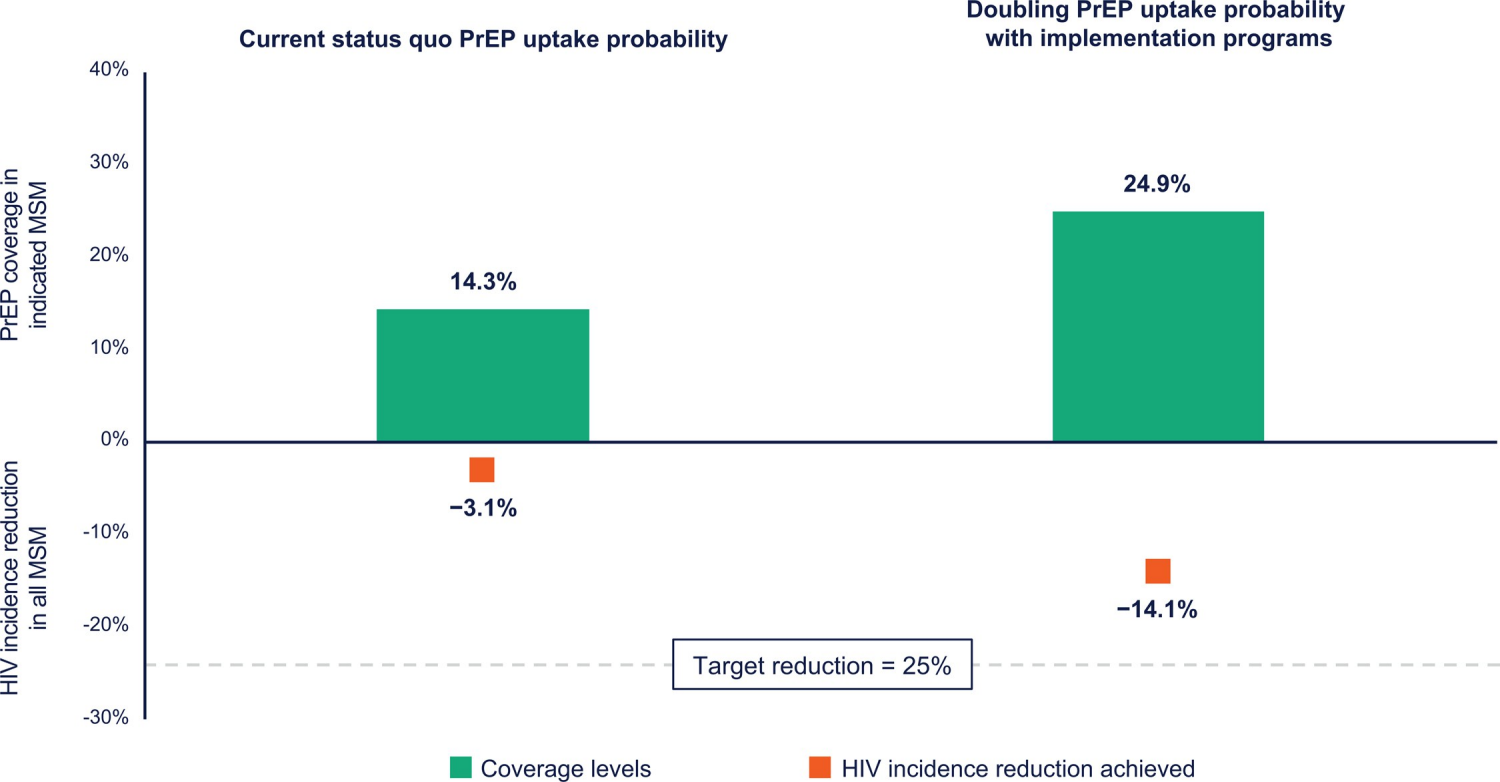
Current PrEP usage trajectory and scenario simulating successful rollout of PrEP implementation programs, presenting the effect of doubling daily-oral PrEP initiations on HIV incidence reduction, in relation to the Georgia Department of Public Health 25% HIV incidence reduction target. HIV, human immunodeficiency virus; PrEP, pre-exposure prophylaxis.

In a scenario simulating successful rollout of implementation support to enhance current daily-oral PrEP programs [14–17], doubling the probability of daily-oral PrEP initiations relative to the reference scenario (status quo) resulted in an increase in PrEP coverage from 14.3% to 24.9%, corresponding to a 14.1% reduction in HIV incidence in the Atlanta MSM community (Fig 1).

**Fig 2:**
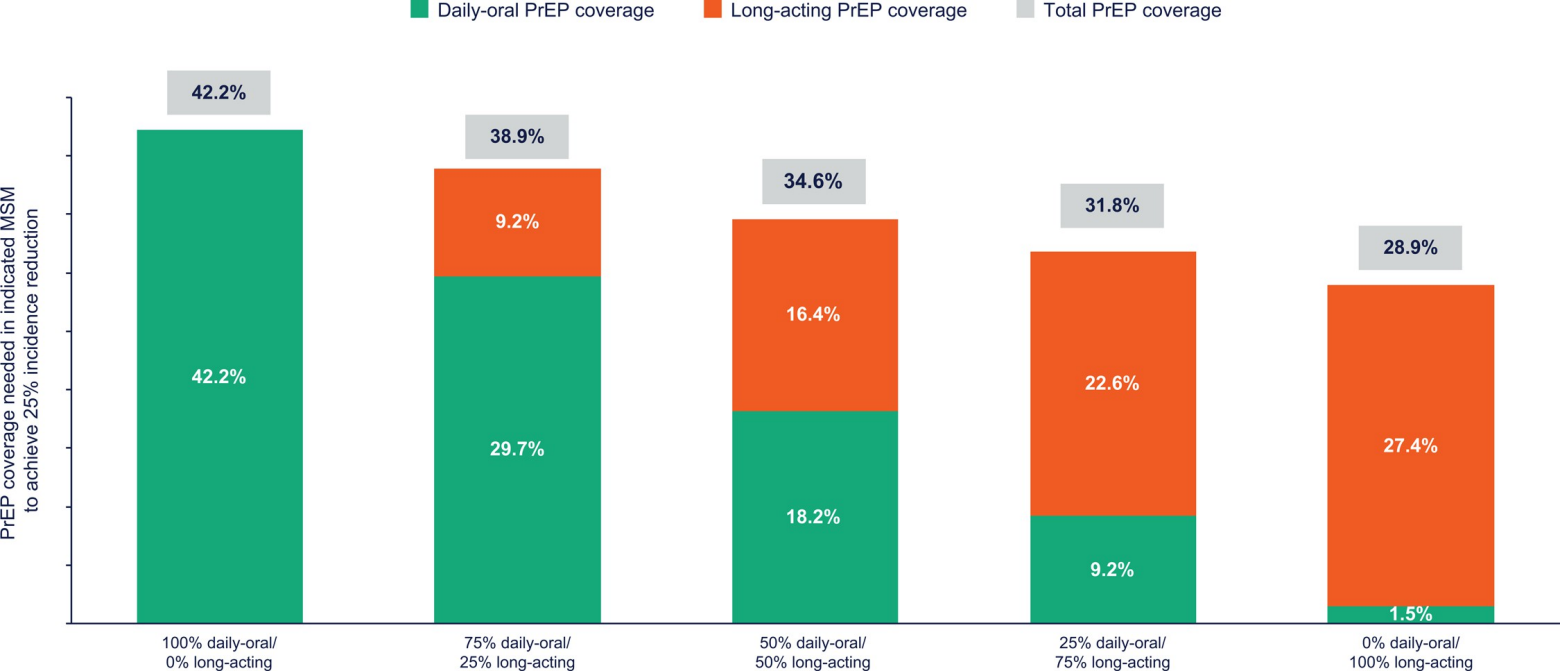
PrEP coverage required to achieve the Georgia Department of Public Health 25% HIV incidence reduction target among MSM in Atlanta, by daily-oral/long-acting CAB PrEP split scenarios. LA, long-acting cabotegravir PrEP; DO, daily oral; HIV, human immunodeficiency virus; PrEP, pre-exposure prophylaxis.

The overall PrEP coverage needed to achieve the target 25% HIV incidence reduction was evaluated across a series of scenarios that incorporated long-acting CAB PrEP, at various shares of usage, into the PrEP modality mix. Without long-acting, 42.2% daily-oral PrEP coverage among indicated MSM in Atlanta would be required to achieve the target 25% HIV incidence reduction in the full MSM community. Due to increased efficacy in preventing HIV acquisition, PrEP coverage required to achieve target HIV incidence reduction decreased progressively with increasing utilization of long-acting CAB PrEP (Fig 2), with 34.6% coverage sufficient to achieve the HIV reduction target in a scenario where half of the users initiated long-acting and the other half daily-oral PrEP. Of note, in a hypothetical scenario where all PrEP users initiated long-acting CAB PrEP, the public health target of 25% HIV incidence reduction was achieved at 28.9% PrEP coverage (Fig 2).

**Fig 3:**
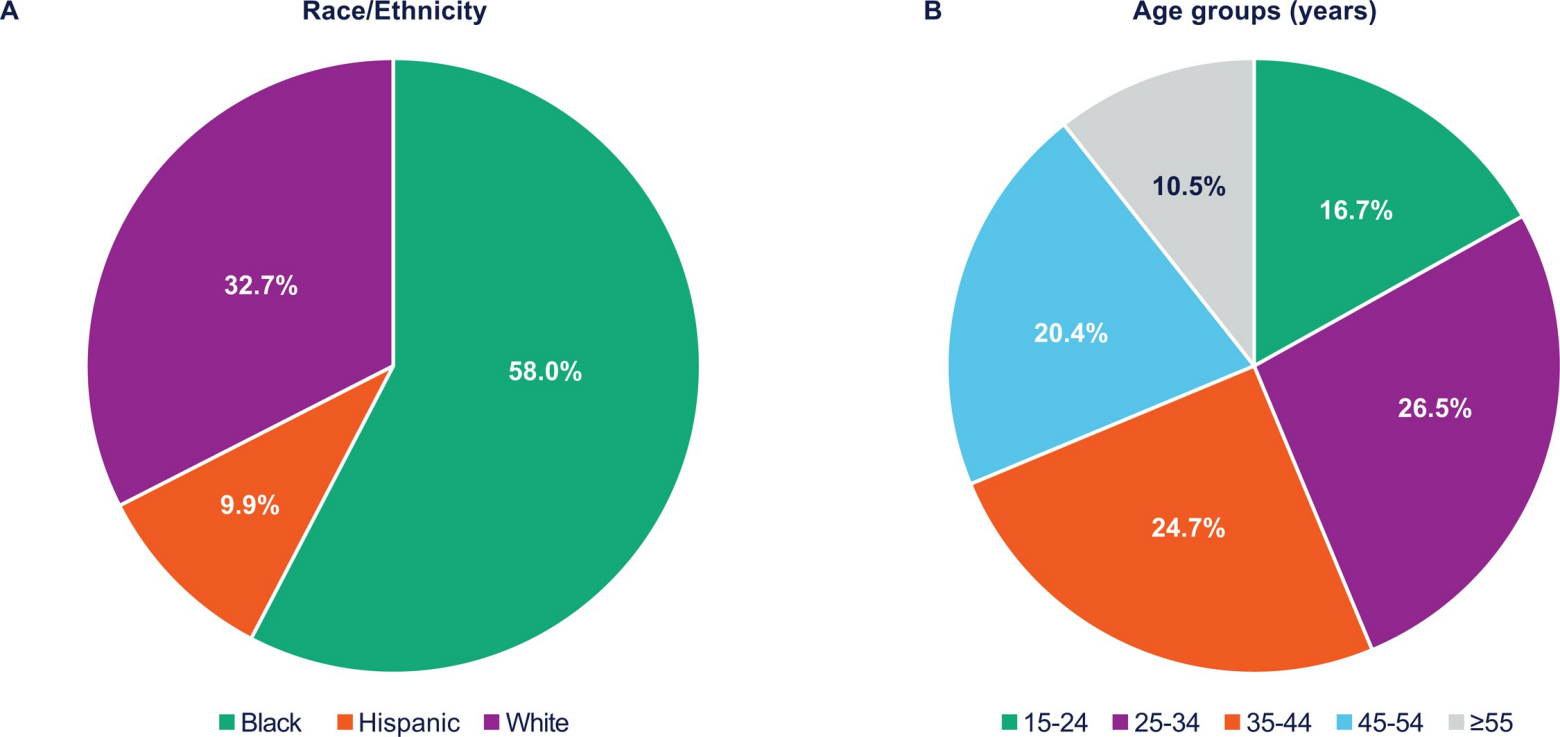
Percentage of HIV infections averted in the 50% daily-oral/50% long-acting CAB PrEP split scenario, by race/ethnicity and age group. CAB, cabotegravir; HIV, human immunodeficiency virus; PrEP, pre-exposure prophylaxis.

Considering the unequal distribution of new HIV diagnoses among racial/ethnic and age groups, the impact of enhancing PrEP coverage on HIV acquisition in different demographic groups was examined. Based on a plausible scenario with 50% of users on daily-oral and 50% on long-acting CAB PrEP, detailed analyses by race/ethnicity and age revealed that most infections were averted in those populations with highest unmet need for prevention; 58.0% of averted infections were in Black/African American MSM (Fig 3A) and 43.2% in MSM aged ≤34 years (Fig 3B). This is despite the fact that this scenario did not differentially target populations based on their demographic characteristics, as PrEP usage was increased across demographic groups proportional to existing levels of use (Table C in S1 Appendix).

In addition to examining the reduction in HIV incidence in the modelled population overall, the reduction in infections in both PrEP users and those who do not use PrEP themselves but indirectly benefit from increased community PrEP coverage was explored. The HIV incidence reduction resulting from an increase in PrEP coverage was evident not only among users of PrEP, but also those in the Atlanta MSM community not engaged with PrEP, who achieved 10.8% HIV incidence reduction in a scenario where 50% of PrEP users received daily-oral PrEP and the other 50% received long-acting CAB PrEP (Table D in S1 Appendix).

## Discussion

In the US, rates of new HIV diagnoses remain high and PrEP coverage remains suboptimal among those who could benefit from PrEP [11], despite the availability of a highly effective daily-oral PrEP option for over a decade and the provision of PrEP free of charge through the federal Ready, Set, PrEP program [36]. To help inform effective public health decision making, this analysis estimated the impact of strategies to increase PrEP coverage levels and resulting decreases in HIV incidence among MSM in Atlanta, GA, through 2030, against targets set by the State of Georgia [28].

The current trajectory of only daily-oral PrEP engagement was projected to result in limited declines in HIV incidence (3.1%), far below the 25% incidence reduction target of the Georgia State Department of Health, indicating additional strategies will be required to achieve targets. Research on implementation programs specifically aimed to increase daily-oral PrEP engagement in key populations by addressing common barriers have demonstrated a doubling of daily-oral PrEP initiations is achievable relative to standard of care [14–17]. A scenario that simulated such a successful rollout of PrEP implementation programs resulted in substantial increases in PrEP coverage from 14.3% to 24.9% in indicated MSM and a corresponding 14.1% decrease in HIV incidence in the full Atlanta MSM community. This finding indicates that resources dedicated to implementation programs with daily-oral PrEP can substantially reduce rates of new HIV diagnoses; however, the limits of what can be achieved with only one PrEP modality is clear as both coverage levels and resulting incidence reductions remained below Georgia’s targets [28].

In addition to implementation programs, strategies to increase PrEP coverage with access to long-acting PrEP will likely be required. Specifically, long-acting CAB PrEP provides superior efficacy in reducing HIV diagnoses, addresses the adherence-dependent efficacy of daily-oral PrEP, supports matching user preference to modality choice, and may increase PrEP engagement similar to increases in contraception use seen following the introduction of long-acting options [18–21].

We examined the PrEP coverage levels needed to achieve the target 25% HIV incidence reduction across a series of scenarios that incorporated a modality mix of daily-oral PrEP and long-acting CAB PrEP at various shares of usage. This analysis found that 42.2% coverage with only daily-oral PrEP would result in achievement of the target HIV incidence reduction. However, this coverage level is substantially beyond the limits found to be attainable with focused implementation programs, which resulted in only 24.9% coverage and indicates that public health targets are likely not achievable with only daily-oral modalities of PrEP.

Increasing the share of long-acting CAB PrEP in the PrEP modality mix decreased the overall coverage levels needed to achieve HIV incidence reduction targets. An overall combined daily-oral and long-acting CAB PrEP coverage of 34.6% (significantly lower than the state’s 50% coverage target) would be sufficient to achieve a 25% reduction in HIV diagnoses if PrEP initiations were distributed equally between the modalities. This suggests long-acting CAB PrEP may be an efficient additional lever to use in PrEP implementation programs, as public health goals with respect to HIV incidence reduction may be achieved at lower, and more readily feasible, overall PrEP coverage levels.

Further analyses found that with achievement of the 25% incidence reduction target in the overall MSM community, the greatest benefit in averted HIV diagnoses was captured by communities disproportionally impacted by HIV and prioritized in national policy, such as young MSM and the Black/African American MSM community. In addition, achievement of the target incidence reductions was estimated to benefit the entire Atlanta MSM community, even those not directly using PrEP, through a reduction in HIV incidence among non-PrEP users of over 10%.

We used a robust, previously validated model which has been recalibrated to reflect recent data on real-world daily-oral PrEP utilization patterns and results from long-acting PrEP trials. The analysis was set among National HIV/AIDS Strategy priority populations and several priority jurisdictions as defined in the EHE strategy [3, 37]. The results presented provide policy makers with various options to achieve their targets at plausible levels of overall PrEP utilization achieved through different PrEP modality mixtures, which may support efficient allocation of limited public health resources. The impact of long-acting PrEP was demonstrated in this evaluation in a key population with barriers to daily-oral PrEP use and in a region with continued high burden of HIV acquisitions and may help inform public health strategies in other key populations and priority geographies with similar characteristics. Specifically, key populations prioritized in public health strategies for whom daily-oral PrEP has not provided significant benefits in the real-world setting, including cisgender and transgender people, may achieve increased rates of PrEP coverage with access to novel PrEP modalities that remove current barriers to use. However, the demographic composition and trajectory of new HIV acquisitions in the Atlanta MSA is unique and further research in additional US geographies is needed to develop strategies that may reduce rates of new HIV acquisitions.

Despite the aforementioned strengths, our analysis also has several limitations. Not all benefits of long-acting CAB PrEP were captured, as the model only assessed PrEP coverage and HIV incidence. Benefits such as potential increased quality of life of PrEP users due to less stigmatization, or reduction in healthcare and societal costs associated with averted HIV infections were not captured within this framework focusing on epidemiological outcomes. Conservatively, the possibility of differential persistence between daily-oral PrEP and long-acting CAB PrEP was not explored in the model. Additionally, to keep the model computationally manageable, cisgender women were not included in the analyses, despite being one of the White House National HIV/AIDS Strategy priority populations [3]. Further research to quantify the rates of HIV acquisition between key populations disproportionally impacted by HIV is needed to evaluate the benefits of PrEP expansion to a broader range of non-PrEP users and key populations than possible in this analysis. Additionally, the demographic composition in the model was matched to the Atlanta MSA with data from the US Census Bureau’s ACS, which represents a sample of the true population and PrEP utilization data from specific trials that may limit the generalizability of the findings. Finally, this analysis did not evaluate the efficiency of PrEP implementation programs if long-acting CAB PrEP use preferentially replaced daily-oral PrEP in individuals with low adherence to daily-oral PrEP, and if PrEP use and coverage was raised in those groups with highest rates of HIV transmission preferentially, potentially resulting in more efficient outcomes.

We used data from real-world daily-oral PrEP studies to inform assumptions on long-acting PrEP uptake and persistence. As real-world long-acting PrEP utilization data become available, further research will be needed. Specifically, if improved real-world persistence rates compared to daily-oral PrEP are reported or long-acting PrEP changes the likelihood of uptake differentially by demographics relative to daily-orals, refined evaluations will be needed to understand the impact on strategies to achieve public health targets.

As the use of long-acting PrEP increases, additional implementation challenges will need to be addressed. The provision of injections may require a reorganization of clinical operation pathways, accounting for additional staff time, frequency of visits and HIV testing, and financial support. While the majority of PrEP is prescribed in specialty care settings, expansion to primary care settings would support continued PrEP scale-up and should be prioritized, as studies have demonstrated that PrEP can be effectively administered beyond specialty care [38, 39]. Additionally, a critical step in PrEP expansion will be identifying additional individuals who can benefit from PrEP, which can be challenged by stigma and discrimination against MSM. For clinicians, the development of trusting and open relationships with patients can support the routine evaluation of sexual history, overcome lack of willingness to take PrEP, and support discussions regarding the various PrEP modality options available to best fit into patients’ lifestyles and preferences [40, 41].

## Conclusions

These results demonstrate that achievement of regional targets for HIV incidence reductions among MSM will require additional strategies to increase engagement with HIV PrEP beyond current levels. Removing barriers to PrEP engagement in key populations through the of expansion of PrEP implementation programs and broadening access to a novel long-acting CAB PrEP modality can support achievement of HIV incidence reductions targets at plausible and efficient levels of PrEP coverage.

## Supporting information

S1 Appendix Appendix for Achieving the State of Georgia 25% HIV incidence reduction target among men who have sex with men in Atlanta through expanded use of multimodal pre-exposure prophylaxis: A mathematical model. (DOCX)
