# Supplementary material for: Achieving the state of Georgia 25% HIV incidence reduction target among men who have sex with men in Atlanta through expanded use of multimodal pre-exposure prophylaxis: A mathematical model
Source: PLoS One. 2025 Jan 9;20(1):e0312369. doi: 10.1371/journal.pone.0312369 (PMC11717278; doi:10.1371/journal.pone.0312369)
Supplement: S1 Appendix — (DOCX) [file pone.0312369.s001.docx]

Appendix for Achieving the State of Georgia 25% HIV incidence reduction target among men who have sex with men in Atlanta through expanded use of multimodal pre-exposure prophylaxis: a mathematical model

## **Model validation**

### Validation of model demographics

The network model was re-estimated from Maloney et al. (2021) [1] in order to align the demographic composition of the agents in the model with that of the Atlanta metropolitan statistical area (MSA) as per the American Community Survey (ACS) 5-year estimate (2018) [2]. The re-estimation required that the model was re-calibrated in terms of the race-specific HIV transmission factors applied in the model. This re-calibration was achieved using an approximate Bayesian computation technique to ensure that the race specific HIV prevalence among men who have sex with men (MSM) after a 60-year burn-in period matched that presented in Rosenberg et al. (2018) [3]. Fig A illustrates the good alignment between the model demographic composition and the census data for the Atlanta MSA.

Fig A. Proportions of model agents by race (top) and age (bottom) vs census data for Atlanta-Sandy Springs-Alpharetta MSA (2018).


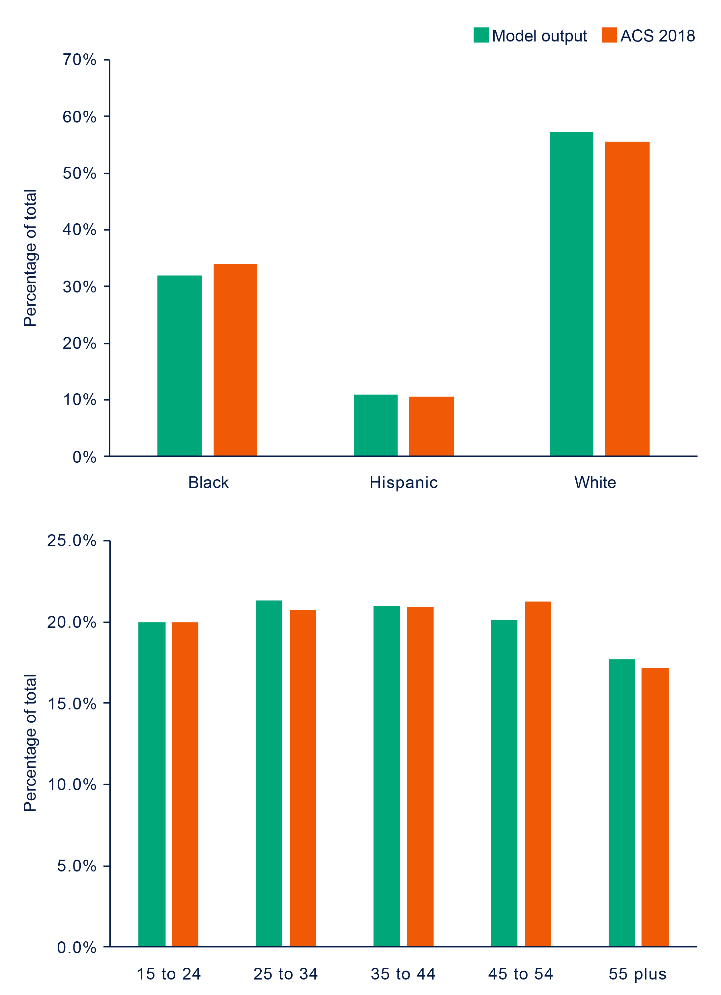


ACS, American Community Survey; MSA, metropolitan statistical area.

### Validation of HIV infections

The model was validated against estimates for the rates of HIV infection incidence and prevalence among MSM for the Atlanta MSA in 2018. Measures of HIV incidence among MSM stratified by age and race are not routinely reported at the level of statistical area by the Centers for Disease Control and Prevention (CDC). Therefore, these were estimated using a combination of data from CDC surveillance reports, US census data and literature estimates of the proportion of MSM in the State of Georgia. The following methodology was employed:

- **Step 1**: The male population by age group was obtained from the 2018 5-year ACS Demographic and Housing Estimates via data.census.gov [2] for the Atlanta-Sandy Springs-Alpharetta MSA. To derive the male populations from the overall, an assumption was made that the ratio of males to females was constant throughout the age categories and reflected that of the over 18 population.
- **Step 2**: The proportions of black of Black, Hispanic, and (by remainder) the White/Other categories were obtained from the same survey and applied to the total male population aged 15 to 64 to generate populations estimates for these categories.
- **Step 3**: The total MSM populations stratified by age for the Atlanta MSA were estimated as follows. Estimates for the absolute numbers of MSM are provided in the Supplement of Jones et al. (2018) [4] for Georgia in 2014. These were divided by the male populations of the respective age category in Georgia in 2014 [5] to obtain the proportion MSM. Because there are differences in the MSM population depending on the urbanicity of the population, an adjustment was made to these proportions by multiplying by a factor of (5.4/3.7), which is the ratio of MSM prevalence in Atlanta MSA compared to Georgia in a study published by Grey et al. 2016 [6]. The proportion MSM in each age category was multiplied by the Atlanta MSA male population size in each age category to obtain the final MSM populations by age.
- **Step 4**: The total MSM in Atlanta MSA was estimated by summing across the age categories from Step 3. The MSM population by race was then estimated by applying the racial composition of the total population. This implicitly assumes that there is no difference in the MSM proportion as a function of race.
- **Step 5**: Using the denominators estimated in Steps 3 and 4, new and existing HIV diagnoses among MSM by age and race were derived from the data supplied in the 2018 CDC surveillance report [7]. Using the diagnoses and proportion of MSM with known HIV status in Fulton County (used as a proxy for Atlanta MSA, derived from Atlas dataset [8]), an estimate of the number of new HIV infections could be made. These were used to derive HIV infection rates per 100 MSM-years and the HIV prevalence for the Atlanta MSA in 2018.
  - These estimates were compared with model predictions for 2018 in Fig B. The model generally agrees with the observed data well, particularly with respect to the overall figures and when broken down by race. There is some deviation in the figures broken down by age, although it is impossible to know whether this reflects an uncertainty in how we have estimated the “real-world” data, or whether it reflects an age pattern of transmission that has not been accurately captured within the model.

Fig B. Model (reference case scenario) predictions of HIV incidence (A, per 100 MSM years) and prevalence (B) by age and race vs estimates derived from surveillance data.


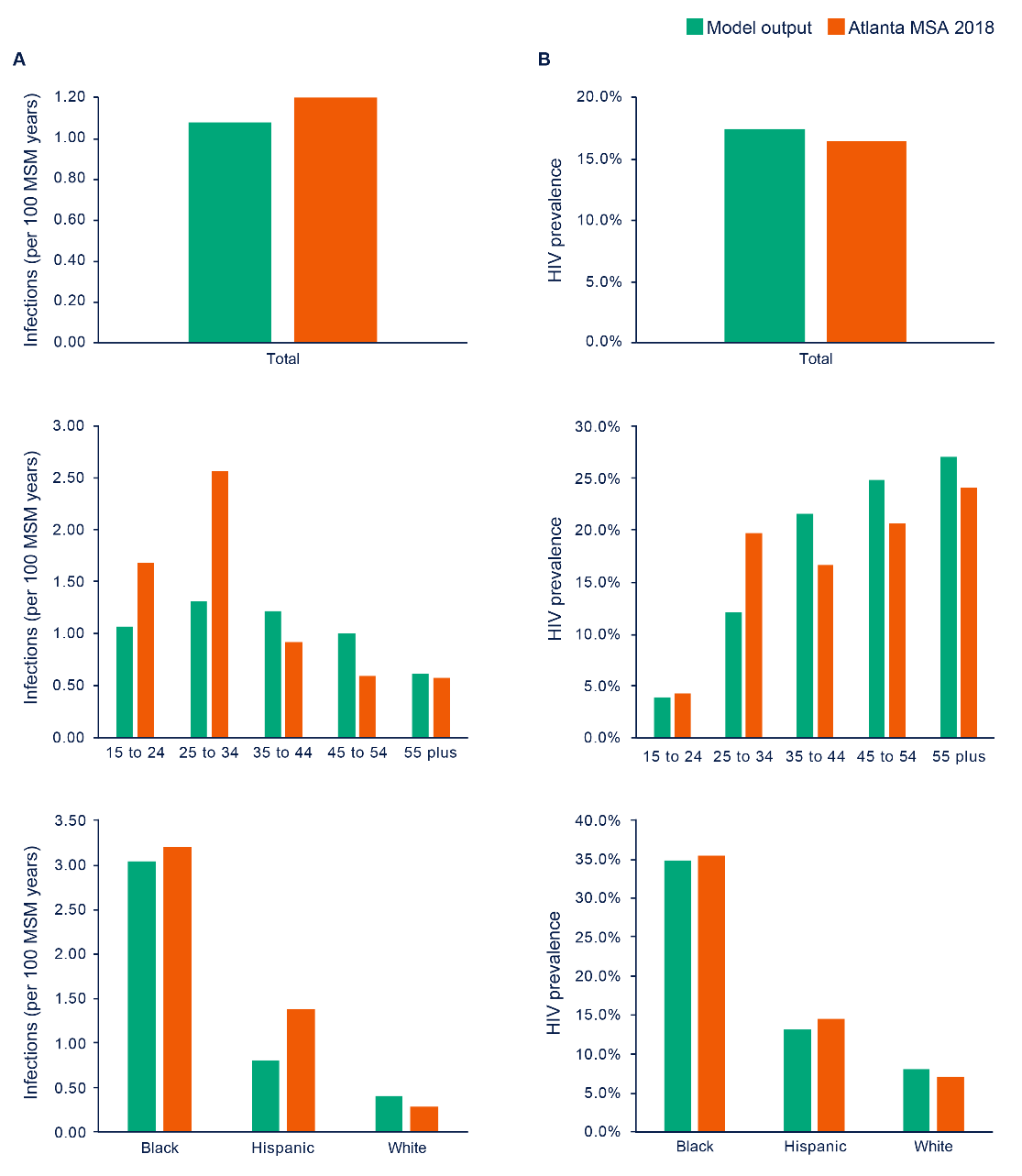


MSA, metropolitan statistical area; MSM, men who have sex with men.

### Validation of pre-exposure prophylaxis uptake

The uptake of pre-exposure prophylaxis (PrEP) in the model was calibrated to data from the CDC Atlas dashboard for Georgia [8]. The probability of initiating PrEP in any given model cycle in a year (2017 to 2021) was adjusted such that the PrEP coverage (defined as the proportion of people with an indication for PrEP who used PrEP) in any calendar year matched the Atlas data for Georgia over the same period.

Fig C shows the model output for the reference case and, because the probability of uptake in any calendar year up to the point we have data available was calibrated to the Atlas data, the agreement is almost exact. The last remaining calibrated uptake probability is then held constant for the model projection period to 2030, and the number of users with at least one prescription during the year levels off at just under 40%.

Fig C. Number of PrEP users with at least one prescription in any calendar year in the state of Georgia as per Atlas CDC data vs. model outputs (reference case scenario).


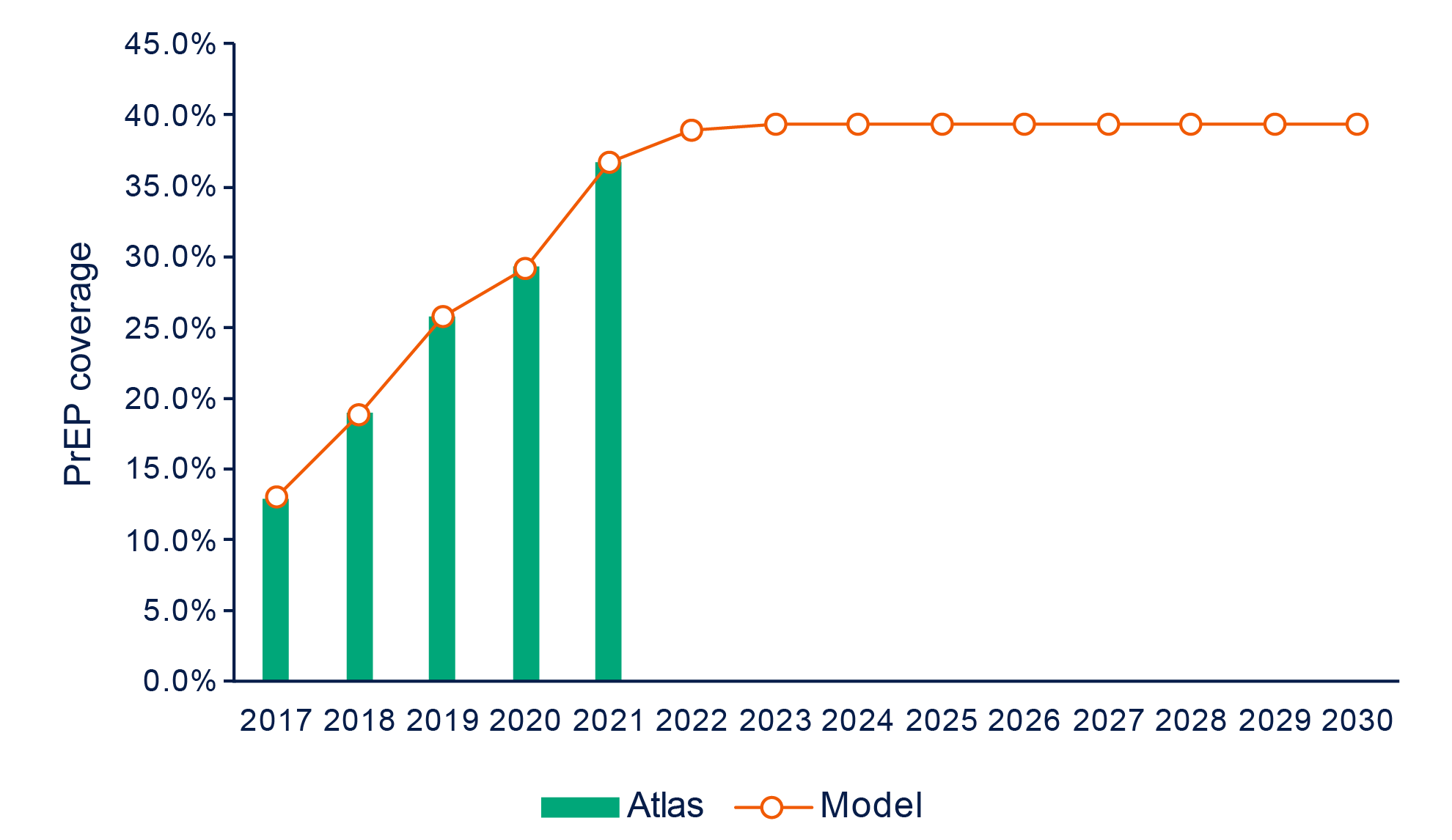


Centers for Disease Control and Prevention; PrEP, pre-exposure prophylaxis.

## **Model updates**

The model used for this analysis was originally developed by Maloney et al. (2021) [1] and a full description of the technical details is included in the supplement of that paper. We made the updates described below to allow additional scenarios and more recent data to be included.

### Adherence

PrEP adherence was treated differently depending on whether the user was on daily oral or long-acting PrEP. For daily oral PrEP, we contacted the authors of Tao et al. (2020) [9], a claims database analysis of 68,788 HIV-1 negative individuals, who initiated tenofovir disoproxil fumarate with emtricitabine (TDF/FTC) for PrEP between January 2017 and December 2019, across payer types in the US. Median age at PrEP initiation was 35 years of age (IQR, 26−43), 88% were men, 70% were white, 14% black and 16% Hispanic. Mean adherence was evaluated by the proportion of days covered and was stratified by age group and race.

For long-acting PrEP, following the literature, trial-based adherence to long-acting injectable (LAI) PrEP (91.5% adherence in HPTN 083) was assumed in the present model. As real-world data on LAI-PrEP adherence becomes available, the model may be updated accordingly.

### PrEP uptake probability

Risk ratios of PrEP uptake by race were taken from the bivariate models presented in Cohen et al. (2015) [10] (white, 1.0; black, 0.76; Hispanic, 0.85). For age, the risk ratios were derived from Atlas CDC database [8] for PrEP users in Georgia. With the 15 to 24 age group set as reference, risk ratios were estimated by taking an average of the ratio of each age group’s proportion of PrEP users to the reference age group over the period 2017-2022 (15 to 24, 1.0; 25 to 34, 2.2; 35 to 44, 3.5; 45 to 54, 3.6; 55plus, 2.4)

### Persistence

Persistence data was based on a claims database analysis including 313,526 HIV-1 negative individuals who initiated TDF/FTC for PrEP between 2012 and 2019, across payer types in the US [9] PrEP users were defined as non-persistent if a gap in prescription fills >30 days was detected. Median age at PrEP initiation was 35 years of age (IQR, 26−43), 88% were men. Among men, median persistence was 118 days (IQR 30−316). Baseline discontinuation rates were derived from this data. In a multivariate analysis, younger age, female sex, and non-white race were associated with higher risk of non-persistence (Table A). In the absence of any real-world data suggesting otherwise, it was assumed that LAI-PrEP persistence was identical to that estimated for daily oral (DO) PrEP.

**Table A. Hazard Ratios For Non-Persistence to DO-PrEP.**

|  | **HR (95% CI)** |
| --- | --- |
| Age at initiation (Reference group: 26-39) | |
| 12-17 | 1.82 (1.74 – 1.90) |
| 18-25 | 1.35 (1.34 – 1.36) |
| 40+ | 0.88 (0.87 – 0.89) |
| Race/Ethnicity (Reference group: White) | |
| Black | 1.11 (1.09 – 1.13) |
| Hispanic | 1.11 (1.09 – 1.13) |
| Asian/Other | 1.06 (1.02 – 1.09) |

CI, confidence interval; DO, daily oral; HR, hazard ratio; PrEP, pre-exposure prophylaxis.

### PrEP efficacy

In the absence of PrEP, per sex-act transmission probability depends on type (insertive or receptive) and is influenced by HIV viral load, acute stage, condom use, and circumcision status [1]. The impact of PrEP on the probability of transmission by act depends on the type of PrEP (LAI versus DO) and, for DO-PrEP, on the level of adherence.

For DO-PrEP, the transmission probability per sex-act was reduced by the incidence rate ratios (IRR) as per the iPrEX study [11] (Table B). Rate ratios were applied in the model depending on the adherence level of the individual.

For LAI PrEP, the efficacy was calculated using the following steps:

- - An adherence-weighted incidence rate ratio for the adherence patterns observed in HPTN 083 for the DO PrEP arm was calculated using the iPrEX study IRRs by adherence level.
  - An estimate of the background (no PrEP) incidence rate in HPTN 083 was made using this adherence-weighted IRR and the HIV incidence rate for those on TDF from HPTN 083.
  - The LAI-PrEP IRR in HPTN 083 could then be estimated by dividing the cabotegravir-LA incidence rate by the estimated background incidence rate.

Following these steps yields an estimated IRR for LAI-PrEP of 0.052 which in the model is assumed to apply uniformly across LAI-PrEP users. This approach will preserve the incidence rate ratio of 0.34 from the trial only when the oral adherence distributions are matched.

**Table B. Incidence Rate Ratios for DO-PrEP Users by Adherence Level.**

| **Adherence level** | **IRR by act with DO-PrEP (Grant 2014 [12], Maloney 2021 [1])** |
| --- | --- |
| Low (< 2 pills per week) | 0.69 |
| Medium (2 to 2 pills per week) | 0.19 |
| High (≥ 4 pills per week) | 0.02 |

DO, daily oral; IRR, incidence rate ratios; PrEP, pre-exposure prophylaxis.

## **Additional results**

### PrEP coverage by demographics

Table C. PrEP Coverage for 50:50 DO vs CAB LA Scenario Required to Achieve Incidence Reduction Target of 25%.

| **PrEP Coverage by Race/Ethnicity and Age** | | | | | |
| --- | --- | --- | --- | --- | --- |
|  | Black | | Hispanic | | White |
| DO PrEP coverage | 14.3% | | 16.4% | | 18.2% |
| LAI PrEP coverage | 13.2% | | 14.8% | | 17.7% |
|  | 15-24 | 25-34 | 35-44 | 45-54 | 55+ |
| DO PrEP coverage | 6.6% | 16.4% | 24.0% | 25.9% | 20.6% |
| LAI PrEP coverage | 6.2% | 14.9% | 21.6% | 23.0% | 18.4% |

CAB LA, cabotegravir long acting; DO, daily oral.

### Incidence rates not on PrEP

Table D. HIV Incidence Rates Among Those Not on PrEP for Reference Scenario and 50:50 DO vs CAB LA Scenario Required to Achieve Incidence Reduction Target of 25%.

| **HIV Infections per 100 PYs by Race/Ethnicity and Age** | | | | | |
| --- | --- | --- | --- | --- | --- |
|  | Black | | Hispanic | | White |
| Reference scenario | 2.77 | | 0.75 | | 0.39 |
| 50:50 DO vs CAB LA | 2.38 | | 0.64 | | 0.31 |
|  | 15-24 | 25-34 | 35-44 | 45-54 | 55+ |
| Reference scenario | 0.90 | 1.20 | 1.20 | 1.05 | 0.64 |
| 50:50 DO vs CAB LA | 0.77 | 1.09 | 1.07 | 0.98 | 0.57 |

CAB LA, cabotegravir long acting; DO, daily oral.

## **References**

1. Maloney KM, Le Guillou A, Driggers RA, Sarkar S, Anderson EJ, Malik AA, et al. Projected impact of concurrently available long-acting injectable and daily-oral human immunodeficiency virus preexposure prophylaxis: a mathematical model. J Infect Dis. 2021;223(1): 72-82.

2. United States Census Bureau. DP05 ACS Demographic and Housing Estimates [Cited 2023 September 21]. Available from: <https://data.census.gov/table?g=310XX00US12060&tid=ACSDP5Y2018.DP05>.

3. Rosenberg ES, Purcell DW, Grey JA, Hankin-Wei A, Hall E, Sullivan PS. Rates of prevalent and new HIV diagnoses by race and ethnicity among men who have sex with men, U.S. states, 2013-2014. Ann Epidemiol. 2018;28(12): 865-73.

4. Jones J, Grey JA, Purcell DW, Bernstein KT, Sullivan PS, Rosenberg ES. Estimating prevalent diagnoses and rates of new diagnoses of HIV at the state level by age group among men who have sex with men in the United States. Open Forum Infect Dis. 2018;5(6): ofy124.

5. United States Census Bureau. Annual Estimates of the Resident Population for Selected Age Groups by Sex for Georgia: April 1, 2010 to July 1, 2019 (SC-EST2019-AGESEX-13). Available from: <https://www2.census.gov/programs-surveys/popest/tables/2010-2019/state/detail/sc-est2019-agesex-13.xlsx>.

6. Grey JA, Bernstein KT, Sullivan PS, Purcell DW, Chesson HW, Gift TL, et al. Estimating the population sizes of men who have sex with men in US states and counties using data from the American community survey. JMIR Public Health Surveill. 2016;2(1): e14.

7. Centers for Disease Control and Prevention. Diagnoses of HIV infection among adults and adolescents in metropolitan statistical areas—United States and Puerto Rico. HIV Surveillance Data Tables 2020;1(3). 2018. Available from: <http://www.cdc.gov/hiv/library/reports/surveillance-data-tables/vol-1-no-3/index.html>.

8. Centers for Disease Control and Prevention. NCHHSTP AtlasPlus. Available from: <https://www.cdc.gov/nchhstp/atlas/index.htm>.

9. Tao L, Shvachko V, Mera R, Das M, Carter C, Magnuson D. 103. Persistence on F/TDF for HIV pre-exposure prophylaxis: insights from real-world evidence. Open Forum Infect Dis. 2020;7(Supplement_1): S181.

10. Cohen SE, Vittinghoff E, Bacon O, Doblecki-Lewis S, Postle BS, Feaster DJ, et al. High interest in preexposure prophylaxis among men who have sex with men at risk for HIV infection: baseline data from the US PrEP demonstration project. J Acquir Immune Defic Syndr. 2015;68(4): 439-448.

11. Grant RM, Lama JR, Anderson PL, McMahan V, Liu AY, Vargas L, et al. Preexposure chemoprophylaxis for HIV prevention in men who have sex with men. N Engl J Med. 2010;363(27): 2587-2599.

12. Grant RM, Anderson PL, McMahan V, Liu A, Amico KR, Mehrotra M, et al. Uptake of pre-exposure prophylaxis, sexual practices, and HIV incidence in men and transgender women who have sex with men: a cohort study. Lancet Infect Dis. 2014;14(9): 820-829.
